# RARE-Bestpractices: a platform for sharing best practices for the management of rare diseases

**DOI:** 10.1186/1750-1172-9-S1-O14

**Published:** 2014-11-11

**Authors:** D Taruscio, C Morciano, P Laricchiuta, P Mincarone, F Palazzo, CG Leo, S Sabina, R Guarino, J Auld, T Sejersen, D Gavhed, K Ritchie, M Hilton-Boon, J Manson, PG Kanavos, D Tordrup, V Tzouma, Y Le Cam, J Senecat, G Filippini, S Minozzi, C Del Giovane, H Schünemann, JJ Meerpohl, B Prediger, L Schell, R Stefanov, G Iskrov, T Miteva-Katrandzhieva, P Serrano-Aguilar, L Perestelo-Perez, MM Trujillo-Martín, J Pérez-Ramos, A Rivero-Santana, A Brand, H van Kranen, K Bushby, A Atalaia, J Ramet, L Siderius, M Posada, I Abaitua-Borda, V Alonso Ferreira, M Hens-Pérez, FJ Manzanares

**Affiliations:** 1 National Centre for Rare Diseases, Istituto Superiore di Sanità, Rome, Italy; 2 National Research Council, Rome, Italy; 3 Jamarau, London, UK; 4 Karolinska Institutet, Stockholm county, Sweden; 5 Healthcare Improvement Scotland, Edinburgh, Scotland; 6 London School of Economics and Political Science, London, UK; 7 EURORDIS, European Organisation for Rare Diseases, Paris, France; 8 Associazione per la Ricerca sull’Efficacia dell’Assistenza Sanitaria Centro Cochrane Italiano, Milan, Italy; 9 University of Freiburg, Freiburg, Germany; 10 Bulgarian Association for Promotion of Education and Science, Plovdiv, Bulgaria; 11 Fundación Canaria de Investigación y Salud, Las Palmas de Gran Canaria, Canary Islands; 12 Universiteit Maastricht, Maastricht, the Netherlands; 13 University of Newcastle Upon Tyne, Newcastle, UK; 14 The European Academy of Paediatrics, Geneva, Switzerland; 15 Institute of Rare Diseases Research, Instituto de Salud Carlos III, Madrid, Spain

## 

Rare diseases; clinical practice guidelines; recommendations

RARE-Bestpractices (http://www.rarebestpractices.eu) is a 4-year project (2013-2016) funded by the EC FP7. The project aims at improving clinical management of patients with rare diseases (RD) and at narrowing the existing gap in quality of healthcare among countries.

## Methods

RARE-Bestpractices (http://www.rarebestpractices.eu) involves 9 EU countries, including 15 partners from academic institutions, governmental bodies, patient organizations and networks, which will exploit the added value of integrating different contributions and viewpoints.

The platform is developed involving both experts in RD research as well as experts in clinical practice guidelines (CPG) and systematic reviews.

## Results

Project expected outputs include: 1) identification of challenges to be considered in deriving high quality standards for CPG on RD; 2) transparent procedures and criteria for the evaluation of CPG and their collection in a publicly searchable database; 3) identification of notation criteria to improve user understandability and implementation of CPG; 4) production of mechanisms to assess RD clinical research needs; 5) development of training activities targeted to key stakeholders to disseminate process and tools for developing and evaluating CPG; 6) the publication of a new scientific journal (http://rarejournal.org)

## Discussion

RARE-Bestpractices addresses the demands from both patients and health care providers for updated and high quality CPG on RD. The project will meet the requirements laid down by to the Directive 2011/24/EU, which endorses EU MS to develop European Reference Networks (ERNs) for RD; in fact, one main criterion for ERNs should be the competence to produce CPG and actively disseminate them among Centers of Expertise.

